# Variance-component-based meta-analysis of gene–environment interactions for rare variants

**DOI:** 10.1093/g3journal/jkab203

**Published:** 2021-06-14

**Authors:** Xiaoqin Jin, Gang Shi

**Affiliations:** State Key Laboratory of Integrated Services Networks, Xidian University, Xi’an 710071, China

**Keywords:** meta-analysis, gene–environment interaction, variance component method, rare variants, whole-exome sequencing, blood pressure, UK Biobank

## Abstract

Complex diseases are often caused by interplay between genetic and environmental factors. Existing gene–environment interaction (G × E) tests for rare variants largely focus on detecting gene-based G × E effects in a single study; thus, their statistical power is limited by the sample size of the study. Meta-analysis methods that synthesize summary statistics of G × E effects from multiple studies for rare variants are still limited. Based on variance component models, we propose four meta-analysis methods of testing G × E effects for rare variants: HOM-INT-FIX, HET-INT-FIX, HOM-INT-RAN, and HET-INT-RAN. Our methods consider homogeneous or heterogeneous G × E effects across studies and treat the main genetic effect as either fixed or random. Through simulations, we show that the empirical distributions of the four meta-statistics under the null hypothesis align with their expected theoretical distributions. When the interaction effect is homogeneous across studies, HOM-INT-FIX and HOM-INT-RAN have as much statistical power as a pooled analysis conducted on a single interaction test with individual-level data from all studies. When the interaction effect is heterogeneous across studies, HET-INT-FIX and HET-INT-RAN provide higher power than pooled analysis. Our methods are further validated via testing 12 candidate gene–age interactions in blood pressure traits using whole-exome sequencing data from UK Biobank.

## Introduction

With the advancement of high-throughput sequencing technologies (Ansorge 2009), a variety of statistical tests (Li and Leal 2008; Madsen and Browning 2009; Wu *et al.* 2011; Lee *et al.* 2012; Cheng *et al.* 2014; Sun *et al.* 2016; Liu *et al.* 2019) have been developed to identify associations between rare variants and complex diseases or traits. For most complex diseases or traits, thousands of genetic variants identified by genome-wide association studies (GWASs) explain only a small proportion of their heritabilities, leaving much of those heritabilities still unexplained; this phenomenon is known as “missing heritability” (Tong and Hernandez 2020). Variants identified through GWASs are mostly common variants with minor allele frequencies (MAFs) larger than 5%. Studies have demonstrated that rare variants may explain some of the “missing heritability” (Lee *et al.* 2014; Kao *et al.* 2017; Yu *et al.* 2018). For example, two rare variants have been identified to be associated with low-density lipoprotein cholesterol and high-density lipoprotein cholesterol (Igartua *et al.* 2017). Five rare variants have been associated with low systolic blood pressure (SBP) (He *et al.* 2017). On the other hand, complex diseases are often caused by a combination of genetic factors, environmental exposures, and the interplay between them; thus, gene–environment interaction (G × E) may explain some of the “missing heritability” of complex diseases (Lim *et al.* 2020).

Recently, an increasing number of statistical methods have been developed for testing G × E with rare variants. These methods aim to detect individually weak but collectively strong G × E effects in a functional set, typically a gene or a gene region. Tzeng *et al.* (2011) proposed a similarity-based regression approach (SIMreg) to test the G × E of rare variants in which trait similarity is regressed on pairwise genetic similarity. The approach is applicable for both continuous and binary traits and allows testing for main genetic effects, interaction effects, and joint effects. Jiao *et al.* (2013) presented a gene-based G × E test (SBERIA) for case-control studies in which gene-environment correlations are used to filter out variants showing no promising G × E effects. Lin *et al.* (2013) proposed a G × E set association test (GESAT) under generalized linear models and tested the interaction effect between a marker set and an environmental variable for continuous and discrete traits. GESAT assumes the G × E effect to be random and employs a variance component score test in a linear mixed model framework. Chen *et al.* (2014) proposed two G × E tests for rare variants: INT-FIX, which treats main genetic effects as fixed effects, and INT-RAN, which treats main genetic effects as random. Lin *et al.* (2016) proposed the interaction sequence kernel association test (iSKAT) for assessing rare variants by environmental interactions, which is robust to the proportion of causal variants in a gene and the directions of the variants’ interaction effects. Liu *et al.* (2016) developed a unified gene-based G × E test, which filters out variants with little evidence of interaction effects. This method was shown to improve the power of interaction analysis by means of an optimal filtering threshold.

All these methods are designed for the analysis of interactions in a single study, which is often underpowered due to limited sample size. A very large number of samples are indeed necessary for identifying genetic variants, especially for finding G × Es (Smith and Day 1984; McCarthy *et al.* 2008). To overcome this limitation, the sharing of results among consortia on the same disease or trait and meta-analyses that combine the results of multiple studies are routine practices (Panagiotou *et al.* 2013). A meta-analysis that combines summary results rather than individual-level raw data from multiple independent studies offers an increased effective sample size and boosted statistical power (Evangelou and Ioannidis 2013; Hu *et al.* 2013; Shi and Nehorai 2017; Jin and Shi 2019a, b). However, little work has been done on the meta-analysis of G × Es with rare variants. Wang *et al.* (2018) suggested a gene-based meta-analysis with filtering to detect G × E effects in case-control studies. In this approach, a variant-level “meta-filtering” test is first conducted, and meta-analysis techniques are then applied to test gene-based G × E effects on the retained variants.

INT-FIX and INT-RAN are gene-based G × E tests for rare variants in a single study, which were proposed by Chen *et al.* (2014) and are implemented in the rareGE package. In this study, we develop corresponding meta-analysis methods based on INT-FIX and INT-RAN, which are two powerful and accessible methods for testing G × Es at the study level. Our meta-analysis methods combine gene-level summary statistics from studies via INT-FIX or INT-RAN tests and consider the G × E effect to be either homogeneous or heterogeneous across studies. In simulation studies, we have evaluated our methods by examining their null distributions and statistical power. Our methods have been further applied in testing 12 candidate gene–age interactions in blood pressure (BP) regulation (Simino *et al.* 2014) using whole-exome sequencing data from UK Biobank (Zhao *et al.* 2020).

## Methods

### INT-FIX and INT-RAN in individual studies

Suppose that there are K studies in a meta-analysis for testing gene-based G × E effects of rare variants in a region. For the *k*-th study, $n_{k}$ individuals have been sequenced in the region, which has $m_{k}$ variants, and $y_{\mathrm{ki}}$ and $G_{\mathrm{ki}}\mathrm{=(}g_{\mathrm{ki}1},g_{\mathrm{ki}2},\cdots ,g_{\mathrm{ki}m_{k}})$ denote the phenotype and genotypes of individual i$\left(1�i�n_{k}\right)$. Under the additive genetic model, $g_{\mathrm{kij}}\mathrm{=0}$, 1, or 2 are the numbers of minor alleles. The first element of the covariate vector $X_{\mathrm{ki}}\mathrm{=(}X_{\mathrm{ki}1},X_{\mathrm{ki}2},\cdots ,X_{\mathrm{ki,}(p_{k}+1)})$ is the intercept, with a value of 1, and the other $p_{k}$ elements are covariates. The environmental factor $E_{\mathrm{ki}}$ is included as one of the covariates for adjusting its main effect.

Under the assumption of independent observations, consider the following linear mixed model for testing the gene by $E_{\mathrm{ki}}$ interaction as follows:

(1) $$y_{\mathrm{ki}}=X_{\mathrm{ki}}\alpha_{k}+G_{\mathrm{ki}}W_{k1}\beta_{k}+E_{\mathrm{ki}}G_{\mathrm{ki}}W_{k2}\gamma_{k}+\varepsilon_{\mathrm{ki}},$$

where $\varepsilon_{\mathrm{ki}}∼N(0,\sigma_{k}^{2})$ is an error term and $\alpha_{k}={\left(\alpha_{k1},\alpha_{k2},\cdots ,\alpha_{k(p_{k}+1)}\right)}^{T}$ represents the effects of the intercept and covariates. $\beta_{k}={\left(\beta_{k1},\beta_{k2},\ldots ,\beta_{km_{k}}\right)}^{T}$ and $\gamma_{k}={\left(\gamma_{k1},\gamma_{k2},\ldots ,\gamma_{km_{k}}\right)}^{T}$ are the main genetic effects and G × E effects, respectively. Assume that $\gamma_{k}$ has a mean of 0 and a covariance matrix $\tau I_{m_{k}}$, where $I_{m_{k}}$ denotes the $m_{k}\times m_{k}$ identity matrix. $W_{k1}$ and $W_{k2}$ denote the $m_{k}\times m_{k}$ diagonal weight matrices with elements $w_{\mathrm{kj}1}$ and $w_{\mathrm{kj}2}$ ($1�j�m_{k}$), which are the weights of the main genetic effects and G × E effects, respectively.

Let $y_{k}={\left(y_{k1},y_{k2},\ldots ,y_{kn_{k}}\right)}^{T}$ be the phenotype vector, let $E_{k}=\mathrm{diag}\{E_{\mathrm{ki}}\}$ be an $n_{k}\times n_{k}$ diagonal matrix, and let $\varepsilon_{k}={\left(\varepsilon_{k1},\varepsilon_{k2},\cdots ,\varepsilon_{kn_{k}}\right)}^{T}$ be the error vector. The linear mixed model is written in matrix form as

(2) $$y_{k}=X_{k}\alpha_{k}+G_{k}W_{k1}\beta_{k}+E_{k}G_{k}W_{k2}\gamma_{k}+\varepsilon_{k}.$$

The null hypothesis with no interaction effect for any variant in the gene is $H_{0}$: τ=0, and the alternative hypothesis of at least one variant having a nonzero interaction effect is $H_{1}:\tau >0$.

In the INT-FIX method (Chen *et al.* 2014), the main genetic effects are assumed to be fixed effects, and the $\beta_{k}$ are the regression coefficients of the main genetic effects. Under the null hypothesis,

(3) $$y_{k}=X_{k}\alpha_{k}+G_{k}W_{k1}\beta_{k}+\varepsilon_{k},$$

where $\mathrm{Var}(\varepsilon_{k})=V=\mathrm{diag}\{\sigma_{k}^{2}\}$. By means of maximum likelihood or restricted maximum likelihood functions, $\alpha_{k}$ and $\beta_{k}$ can be estimated; the corresponding estimates are denoted by ${\hat{\alpha }}_{\mathrm{kF}}$ and ${\hat{\beta }}_{\mathrm{kF}}$, respectively. Then, the estimated mean and covariance matrix of $y_{k}$ are

$${\hat{\mu }}_{\mathrm{kF}}=X_{k}{\hat{\alpha }}_{\mathrm{kF}}+G_{k}W_{k1}{\hat{\beta }}_{\mathrm{kF}},$$

and

$${\hat{V}}_{F}\mathrm{=diag\{}{\hat{\sigma }}_{\mathrm{kF}}^{2}\},$$

where ${\hat{\mu }}_{\mathrm{kF}}={\left({\hat{\mu }}_{k1F},{\hat{\mu }}_{k2F},\cdots ,{\hat{\mu }}_{kn_{k}F}\right)}^{T}$.

The INT-FIX statistics of testing the G × E interaction is

(4) $$Q_{k\mathrm{,INT-FIX}}=\sum_{j\mathrm{=1}}^{m_{k}}w_{\mathrm{kj}2}^{2}S_{\mathrm{kj}\mathrm{,INT-FIX}}^{2},$$

where

(5) $$S_{\mathrm{kj}\mathrm{,INT-FIX}}=\sum_{i\mathrm{=1}}^{n_{k}}\frac{E_{\mathrm{ki}}g_{\mathrm{kij}}(y_{\mathrm{ki}}-{\hat{\mu }}_{\mathrm{kiF}})}{{\hat{\sigma }}_{\mathrm{kF}}^{2}}$$

is the G × E score statistic for the *j*-th variant. The asymptotic distribution of $Q_{k\mathrm{,INT-FIX}}$ is a mixture of chi-square distributions, and the *P*-value can be calculated via Kuonen’s saddlepoint method (Kuonen 1999; Chen *et al.* 2014).

In the INT-RAN method (Chen *et al.* 2014), the main genetic effects are assumed to be random, and the elements of $\beta_{k}$ follow a normal distribution with zero mean and covariance matrix $\delta I_{m_{k}}$. Under the null hypothesis, the working model is written as (3), with $\mathrm{Var}(y_{k})=\delta G_{k}W_{k1}W_{k1}G_{k}^{T}+\sigma_{k}^{2}I_{n_{k}}$, and ${\hat{\alpha }}_{\mathrm{kR}}$, ${\hat{\delta }}_{R}$, and ${\hat{\sigma }}_{\mathrm{kR}}^{2}$ can be numerically estimated by means of maximum likelihood or restricted maximum likelihood functions (Chen *et al.* 2014). The estimated mean and covariance matrix of $y_{k}$ are

$${\hat{\mu }}_{\mathrm{kR}}=X_{k}{\hat{\alpha }}_{\mathrm{kR}}+G_{k}W_{k1}{\hat{\beta }}_{\mathrm{kR}}$$

$${\hat{V}}_{\mathrm{kR}}={\hat{\delta }}_{R}G_{k}W_{k1}W_{k1}G_{k}^{T}+{\hat{\sigma }}_{\mathrm{kR}}^{2}I_{n_{k}}$$

where ${\hat{\mu }}_{\mathrm{kR}}={\left({\hat{\mu }}_{k1R},{\hat{\mu }}_{k2R},\cdots ,{\hat{\mu }}_{kn_{k}R}\right)}^{T}$ and ${\hat{\beta }}_{\mathrm{kR}}={\hat{\delta }}_{R}W_{k1}G_{k}^{T}{\hat{V}}_{\mathrm{kR}}^{-1}(y_{k}-X_{k}{\hat{\alpha }}_{\mathrm{kR}})$.

The INT-RAN test statistic is

(6) $$Q_{k\mathrm{,INT-RAN}}=\sum_{j\mathrm{=1}}^{m_{k}}w_{\mathrm{kj}2}^{2}S_{\mathrm{kj}\mathrm{,INT-RAN}}^{2},$$

where

(7) $$S_{\mathrm{kj}\mathrm{,INT-RAN}}=\sum_{i\mathrm{=1}}^{n_{k}}\frac{E_{\mathrm{ki}}g_{\mathrm{kij}}(y_{\mathrm{ki}}-{\hat{\mu }}_{\mathrm{kiR}})}{{\hat{\sigma }}_{\mathrm{kR}}^{2}}$$

is the G × E score statistic for the *j*-th variant. The $Q_{k\mathrm{,INT-RAN}}$ statistic asymptotically follows a mixture of chi-square distributions, and the corresponding *P*-value can again be computed via Kuonen’s saddlepoint method (Kuonen 1999; Chen *et al.* 2014).

### Meta-analysis of summary statistics from INT-FIX and INT-RAN

The meta-analysis of G × E effects is performed based on summary statistics from each study. The required summary statistics for each study include the MAFs of all variants, the G × E score statistic (5) or (7) for each variant and the regional G × E relationship matrix (Zhang and Lin 2003; Wu *et al.* 2011).

For the meta-analysis results of INT-FIX, the regional G × E relationship matrix of the *k*-th study is

(8) $$\Phi_{\mathrm{kF}}=W_{k2}G_{k}^{T}E_{k}({\hat{V}}_{\mathrm{kF}}^{-1}-{\hat{V}}_{\mathrm{kF}}^{-1}Z_{k}{(Z_{k}^{T}{\hat{V}}_{\mathrm{kF}}^{-1}Z_{k})}^{-1}Z_{k}^{T}{\hat{V}}_{\mathrm{kF}}^{-1})E_{k}G_{k}W_{k2},$$

where $Z_{k}={\left(Z_{k1},Z_{k2},\ldots ,Z_{kn_{k}}\right)}^{T}$ and $Z_{\mathrm{ki}}=(X_{\mathrm{ki}},G_{\mathrm{ki}}W_{k1})$.

For INT-RAN, the regional G × E relationship matrix of the *k*-th study is

(9) $$\Phi_{\mathrm{kR}}=W_{k2}G_{k}^{T}E_{k}({\hat{V}}_{\mathrm{kR}}^{-1}-{\hat{V}}_{\mathrm{kR}}^{-1}X_{k}{\left(X_{k}^{T}{\hat{V}}_{\mathrm{kR}}^{-1}X_{k}\right)}^{-1}X_{k}^{T}{\hat{V}}_{\mathrm{kR}}^{-1})E_{k}G_{k}W_{k2}.$$

For convenience, we assume that all variants can be observed in each of the K studies, that is, $m_{1}=m_{2}=\cdots =m_{K}=m$. When some variant is monomorphic in a study, its G × E score statistic and corresponding elements in the relationship matrices can be simply set to zero.

We consider two types of meta-analyses, under scenarios in which the G × E effects are homogeneous and heterogeneous across different studies. For the scenario in which the G × E effects are homogeneous across the different studies, we propose the following G × E test statistics for the meta-analysis of summary statistics from INT-FIX and INT-RAN:

(10) $$Q_{\mathrm{HOM-INT-FIX}}=\sum_{j\mathrm{=1}}^{m}(\sum_{k\mathrm{=1}}^{K}w_{\mathrm{kj}2}S_{\mathrm{kj}\mathrm{,INT-FIX}}{)}^{2},$$

(11) $$Q_{\mathrm{HOM-INT-RAN}}=\sum_{j\mathrm{=1}}^{m}(\sum_{k\mathrm{=1}}^{K}w_{\mathrm{kj}2}S_{\mathrm{kj}\mathrm{,INT-RAN}}{)}^{2}.$$

Similar to the meta-analysis of homogeneous main genetic effects presented in Lee *et al.* (2013), these two statistics first combine the weighted G × E score statistics across different studies for each variant and then aggregate the squared score statistics of all variants in a gene. Here, the weights are based on the MAFs of the variants estimated across all studies. Under the null hypothesis, $Q_{\mathrm{HOM-INT-FIX}}$ asymptotically follows a mixed 1-df chi-square distribution (Lee *et al.* 2013; Chen *et al.* 2014), namely, $Q_{\mathrm{HOM-INT-FIX}}∼\sum_{j\mathrm{=1}}^{m}\lambda_{j}\chi_{j\mathrm{,1}}^{2}$, where $\lambda_{1},\lambda_{2},\cdots ,\lambda_{m}$ are the nonzero eigenvalues of

(12) $$\Psi_{\mathrm{INT-FIX}}=\sum_{k\mathrm{=1}}^{K}W_{k2}\Phi_{\mathrm{kF}}W_{k2},$$

and the *P*-value can be calculated via Kuonen’s saddlepoint method (Kuonen 1999). Similarly, we have the test statistic $Q_{\mathrm{HOM-INT-RAN}}∼\sum_{j\mathrm{=1}}^{m}\lambda_{j}\chi_{j\mathrm{,1}}^{2}$, where $\lambda_{1},\lambda_{2},\cdots ,\lambda_{m}$ are the nonzero eigenvalues of

(13) $$\Psi_{\mathrm{INT-RAN}}=\sum_{k\mathrm{=1}}^{K}W_{k2}\Phi_{\mathrm{kR}}W_{k2}.$$

For the scenario in which the G × E effects are assumed to be heterogeneous across the different studies, we propose the following G × E test statistics for the meta-analysis of the summary statistics from INT-FIX and INT-RAN:

(14) $$Q_{\mathrm{HET-INT-FIX}}=\sum_{j\mathrm{=1}}^{m}\sum_{k\mathrm{=1}}^{K}w_{\mathrm{kj}2}^{2}S_{\mathrm{kj,}\mathrm{INT-FIX}}^{2},$$

(15) $$Q_{\mathrm{HET-INT-RAN}}=\sum_{j\mathrm{=1}}^{m}\sum_{k\mathrm{=1}}^{K}w_{\mathrm{kj}2}^{2}S_{\mathrm{kj,}\mathrm{INT-RAN}}^{2}.$$

As in the meta-analysis of heterogeneous main genetic effects presented in Lee *et al.* (2013), these test statistics aggregate the squared and weighted score statistics across all studies and all variants. In HET-INT-FIX and HET-INT-RAN, the weights are based on the study-specific MAFs. Here, $Q_{\mathrm{HET-INT-FIX}}∼\sum_{j\mathrm{=1}}^{m}\lambda_{j}\chi_{j\mathrm{,1}}^{2}$, where $\lambda_{1},\lambda_{2},\cdots ,\lambda_{m}$ are the nonzero eigenvalues of $\Psi_{\mathrm{INT-FIX}}$, and $Q_{\mathrm{HET-INT-RAN}}∼\sum_{j\mathrm{=1}}^{m}\lambda_{j}\chi_{j\mathrm{,1}}^{2}$, where $\lambda_{1},\lambda_{2},\cdots ,\lambda_{m}$ are the nonzero eigenvalues of $\Psi_{\mathrm{INT-RAN}}$. The *P*-values of the mixed $\chi^{2}$s can also be calculated via Kuonen’s saddlepoint method (Kuonen 1999).

For trans-ethnic meta-analyses, we assume that the G × E effects in studies of the same ancestry are homogeneous, and heterogeneous for studies of different ancestries. Suppose that there are K studies from B ancestries. In the b-th ancestry, there are $k_{b}$ studies, b=1,2,…,B. Denote ${\tilde{K}}_{b}=k_{1}+k_{2}+\cdots +k_{b}$ as the number of studies from ancestry 1 to b, in particular, ${\tilde{K}}_{0}=0$. We propose the following G × E test statistics for the trans-ethnic meta-analysis of the summary statistics from INT-FIX and INT-RAN:

(16) $$Q_{\mathrm{HET-INT-FIX}}=\sum_{j\mathrm{=1}}^{m}\sum_{b\mathrm{=1}}^{B}{\left(\sum_{k={\tilde{K}}_{b-1}+1}^{{\tilde{K}}_{b}}w_{\mathrm{kj}2}S_{kj,\text{INT}-\text{FIX}}\right)}^{2},$$

(17) $$Q_{\mathrm{HET-INT-RAN}}=\sum_{j\mathrm{=1}}^{m}\sum_{b\mathrm{=1}}^{B}{\left(\sum_{k={\tilde{K}}_{b-1}+1}^{{\tilde{K}}_{b}}w_{\mathrm{kj}2}S_{kj,\text{INT}-\text{RAN}}\right)}^{2}.$$

The test statistics combine the weighted score statistic of the j-th variant from studies of the same ancestry, then aggregate the squared scores across all ancestries and all variants. Here, the weights are based on ancestry-specific MAFs. When all studies in the meta-analysis are of the same ancestry, Equations (16) and (17) reduce to Equations (10) and (11), respectively. When all studies have different ancestries, Equations (16) and (17) reduce to Equations (14) and (15), respectively.

### Data availability

This research has been conducted using the UK Biobank Resource under Application Number 44080. Corresponding R codes for meta-analysis methods of testing G × E effects in this article are available at GitHub: https://github.com/jlyx53/Codes-for-Meta-analyses-of-G-E-effects.

## Results

### Numerical simulations

We conducted simulation studies to evaluate the null distributions and statistical power of our four meta-statistics for a continuous phenotype. We considered three scenarios as summarized in Table 1. Each has three studies with sample sizes of 1600, 2200, and 3200, referred to study 1, study 2, and study 3, respectively. For scenario 1, all studies are European (EUR) samples and have the same set of covariates; For scenario 2, three studies are all EUR samples but have different set of covariates; For scenario 3, three studies have different set of covariates and have different ancestries: the first two studies are EUR samples and the third study is African-American (AA) samples. In the power analysis, for each scenario, we considered cases with homogeneous and heterogeneous G × E effects across studies, and also five different levels of main and interaction effects.

**Table 1:** Simulation study settings for three scenarios

Scenario	Pop	Sample sizes			Covariates		
		Study 1	Study 2	Study 3	Study 1	Study 2	Study 3
1	EUR	1,600	2,200	3,200	(BMI, age, gender)	(BMI, age, gender)	(BMI, age, gender)
2	EUR	1,600	2,200	3,200	(BMI)	(BMI, age)	(BMI, age, gender)
3	EUR+AA	1,600	2,200	3,200	(BMI)	(BMI, age)	(BMI, age, gender)

Pop, the populations of three studies. EUR, refers to all three studies are European samples. EUR+AA, refers to study 1 and study 2 are European samples and study 3 are African-American samples.

Using the calibrated coalescent model implemented in COSI (Schaffner *et al.* 2005), we first simulated 10,000 EUR haplotypes and 10,000 AA haplotypes over a 200 kb region. The simulation parameters were set to mimic the linkage disequilibrium structure, local recombination rate and population history of the EUR and AA populations (Lee *et al.* 2013). Then, for scenarios 1 and 2, we randomly paired the EUR haplotypes to obtain diploid genotype data of 10,000 EUR individuals and randomly selected 7000 out of the 10,000 individuals. For scenario 3, we randomly paired the EUR haplotypes to obtain diploid genotype data of 10,000 individuals and randomly selected 3800 out of them. Similarly, we obtained diploid genotype data of 10,000 AA individuals, out of which we randomly selected 3200 individuals. Since the average exon length of a gene is approximately 3 kb (Pruitt *et al.* 2012), we randomly selected a 3 kb subregion within the 200 kb region for each replicate of the genotype data and retained variants with MAFs less than 0.03. We repeated this process 1000 times and generated 1000 replicates of genotype data sets for the three scenarios. On average, there were 54 rare variants in the 3 kb region under scenarios 1 and 2 and 87 under scenario 3. For scenarios 1 and 2, out of the genotype data of the 7000 EUR individuals, we randomly selected 1600, 2200, and 3200 for the samples of study 1, study 2, and study 3, respectively. For scenario 3, out of the genotype data of the 3800 EUR individuals, we randomly selected 1600 and 2200 for the samples of study 1 and study 2, respectively.

To evaluate the null distributions of the proposed meta-analysis statistics, we generated phenotype data sets under the null model. To reduce the computational burden, we simulated 20 replicates of covariates and phenotype for each of the 1000 genotype sets, which yielded 20,000 genotype-phenotype data sets. For scenario 1, the continuous phenotypes for the k-th (k=1,2,3) study were generated by means of the following linear model:

(18) $$y_{k}\mathrm{=0}\mathrm{.5}\mathrm{se}x_{k}\mathrm{+0}\mathrm{.05}\mathrm{ag}e_{k}\mathrm{+0}\mathrm{.1}\mathrm{bm}i_{k}+\varepsilon_{k},$$

where $\mathrm{se}x_{k}\mathrm{=(}\mathrm{se}x_{k1},\cdots ,\mathrm{se}x_{kn_{k}}{)}^{T}$ is a binary covariate vector in which each element $\mathrm{se}x_{\mathrm{ki}}$ is drawn from a Bernoulli distribution with probability 0.5; $\mathrm{ag}e_{k}\mathrm{=(}\mathrm{ag}e_{k1},\cdots ,\mathrm{ag}e_{kn_{k}}{)}^{T}$ and $\mathrm{bm}i_{k}\mathrm{=(}\mathrm{bm}i_{k1},\cdots ,\mathrm{bm}i_{kn_{k}}{)}^{T}$ are continuous covariate vectors in which each element is normally distributed, $\mathrm{ag}e_{\mathrm{ki}}\mathrm{∼N(50,}5^{2})$ and $\mathrm{bm}i_{\mathrm{ki}}\mathrm{∼N(25,}4^{2})$, for $1�i�n_{k}$; and $\varepsilon_{k}\mathrm{=(}\varepsilon_{k1},\cdots ,\varepsilon_{kn_{k}}{)}^{T}$ represents the random errors, with each element following a standard normal distribution. For scenarios 2 and 3, we generated the phenotypes of study 1 according to Equation (18) by removing the age and gender effects, and generated the phenotypes of study 2 by removing the gender effect. The phenotypes of study 3 were generated with the full covariates. Under the null hypothesis, the genotypes are not associated with the phenotype.

To evaluate the statistical power of our proposed meta-analysis methods, we generated phenotypes under the alternative model based on the 1000 genotype data sets described previously. For each of the three scenarios, we considered five different levels of genetic main and interaction effects: level 1 refers to the existence of only genetic main effects but without interaction effects, level 2 refers to the existence of genetic main effects and weak interaction effects, level 3 refers to the existence of both genetic main effects and interaction effects, level 4 refers to the existence of interaction effects and weak genetic main effects, level 5 refers to the existence of only interaction effects but without genetic main effects. For each of the five levels, we assumed that 20, 40, or 60% of the rare variants were causal variants. We simulated one replicate of phenotype and covariates for each genotype data set. The covariates followed the same distributions described previously. For scenario 1, the phenotypes for the k-th study were generated by means of the following linear model:

(19) $$y_{k}\mathrm{=0}\mathrm{.5}\mathrm{se}x_{k}\mathrm{+0}\mathrm{.05}\mathrm{ag}e_{k}\mathrm{+0}\mathrm{.1}\mathrm{bm}i_{k}+G_{k}W_{1k}\beta_{k}+E_{k}G_{k}W_{2k}\gamma_{k}+\varepsilon_{k},$$

where $G_{k}$ is the genotype matrix of the causal variants in study k and $\beta_{k}$ has elements $\beta_{\mathrm{kj}}\mathrm{∼N(0,}\delta_{1}^{2})$. We used body mass index (BMI) as the environmental factor $E_{k}\mathrm{=diag\{}E_{\mathrm{ki}}\}$, which was centered to have a mean of 0. $\gamma_{k}$ represents the effects of gene–BMI interaction, with elements $\gamma_{\mathrm{kj}}\mathrm{∼N(0,}\tau_{1}^{2})$. For scenarios 2 and 3, partial covariates were used as described in Table 1. The values of $\delta_{1}$ and $\tau_{1}$ for the five levels of genetic main and interaction effects were: level 1 with $\delta_{1}=0.2,\tau_{1}=0$, level 2 with $\delta_{1}=0.2,\tau_{1}=0.005$, level 3 with $\delta_{1}=0.2,\tau_{1}=0.05$, level 4 with $\delta_{1}=0.002,\tau_{1}=0.05$, level 5 with $\delta_{1}=0,\tau_{1}=0.05$.

For the case of homogeneous variant and interaction effects across studies, under all the three scenarios, we simulated the same variant effects and gene-BMI interaction effects across all studies, that is, $\beta_{1}=\beta_{2}=\cdots =\beta_{K}=\beta$ and $\gamma_{1}=\gamma_{2}=\cdots =\gamma_{K}=\gamma$. For the case of heterogeneous variant and interaction effects across studies, under scenarios 1 and 2, we simulated the variant effects and gene-BMI interaction effects for each study independently. Under scenario 3, we simulated the same variant effects and gene-BMI interaction effects for study 1 and study 2 that have the EUR ancestry, and simulated the variant effects and gene-BMI interaction effects for study 3 with AA ancestry separately.

In all simulations, the variant weights followed Beta distributions, $w_{j}\mathrm{∼Beta(MA}F_{j}\mathrm{,1,25)}$, as suggested by Wu *et al.* (2011), where j denotes the MAF of variant j. For scenarios 1 and 2, $\mathrm{MA}F_{j}$ was estimated based on all studies in HOM-INT-FIX and HOM-INT-RAN and was specific to each study in HET-INT-FIX and HET-INT-RAN. For scenario 3, $\mathrm{MA}F_{j}$ was estimated based on all studies in HOM-INT-FIX and HOM-INT-RAN and was estimated based on ancestry-specific studies in HET-INT-FIX and HET-INT-RAN. The gene-BMI interaction was considered significant if its *P*-value was less than the significance level of $2.5\times 10^{-6}$, and empirical power was calculated as the proportion of significant results among 1000 replicates.

### Null distributions of the meta-statistics

We examined the distributions of our proposed HOM-INT-FIX, HOM-INT-RAN, HET-INT-FIX, and HET-INT-RAN meta-statistics using the data sets simulated under the null hypothesis. We first computed the G × E score statistics of each study according to formulas (5) and (7) and the regional G × E relationship matrix of each study according to formulas (8) and (9). Then, for scenarios 1 and 2, we combined the study-level G × E score statistics using formulas (10)–(11) and (14)–(15) to obtain the four meta-statistics. For scenario 3, we combined the study-level G × E score statistics using formulas (10)–(11) and (16)–(17). We computed the regional G × E relationship matrices for the meta-analysis by combining the regional relationship matrices from each study according to Equations (12) and (13). The *P*-values of the four meta-statistics were calculated according to their theoretical distributions and Kuonen’s saddlepoint method. The distributions of the empirical *P*-values under the null hypothesis were compared with the expected uniform distribution between 0 and 1.

**Figure 1:**
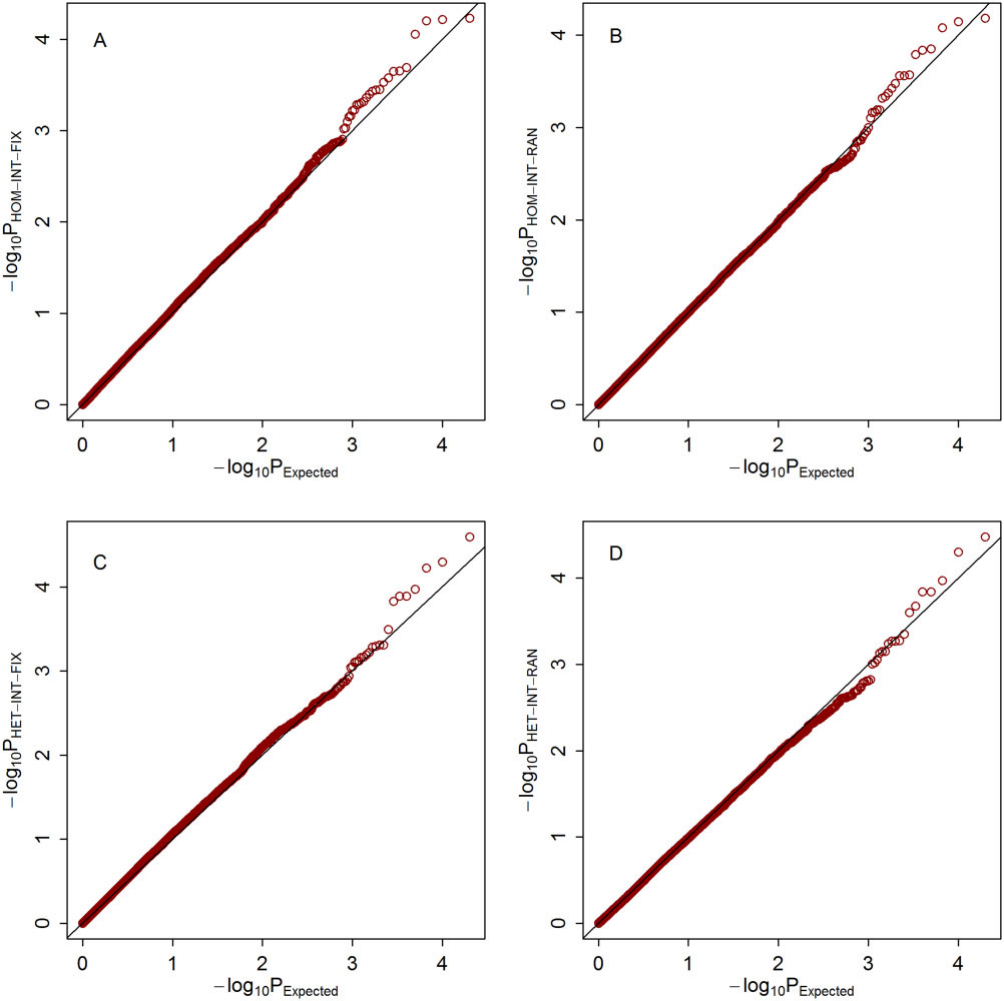
Q–Q plots of the null distributions of the meta-statistics under scenario 1. The horizontal axis represents the negative log_10_ of the expected *P*-values, and the vertical axis represents the negative log10 of the observed *P*-values. (A) HOM-INT-FIX; (B) HOM-INT-RAN; (C) HET-INT-FIX; (D) HET-INT-RAN.

**Figure 2:**
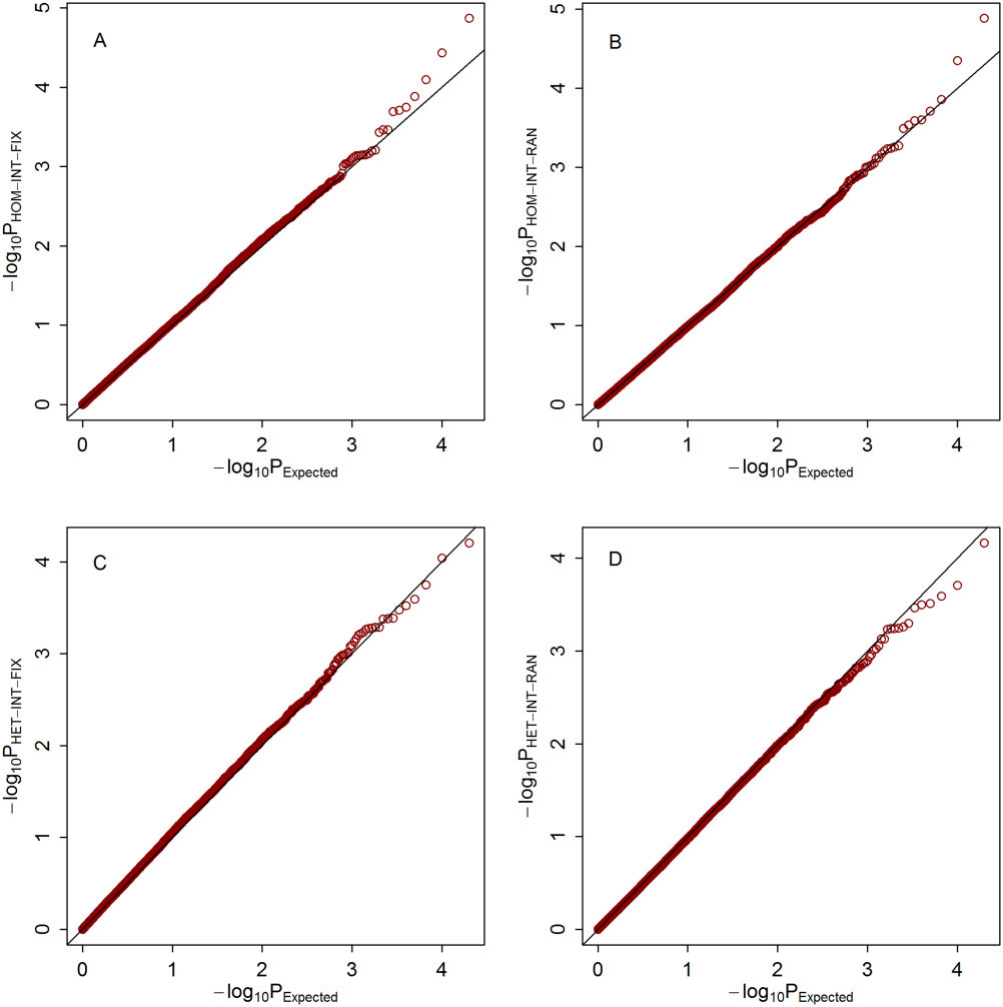
Q–Q plots of the null distributions of the meta-statistics under scenario 2. The horizontal axis represents the negative log10 of the expected *P*-values, and the vertical axis represents the negative log10 of the observed *P*-values. (A) HOM-INT-FIX; (B) HOM-INT-RAN; (C) HET-INT-FIX; (D) HET-INT-RAN.

**Figure 3:**
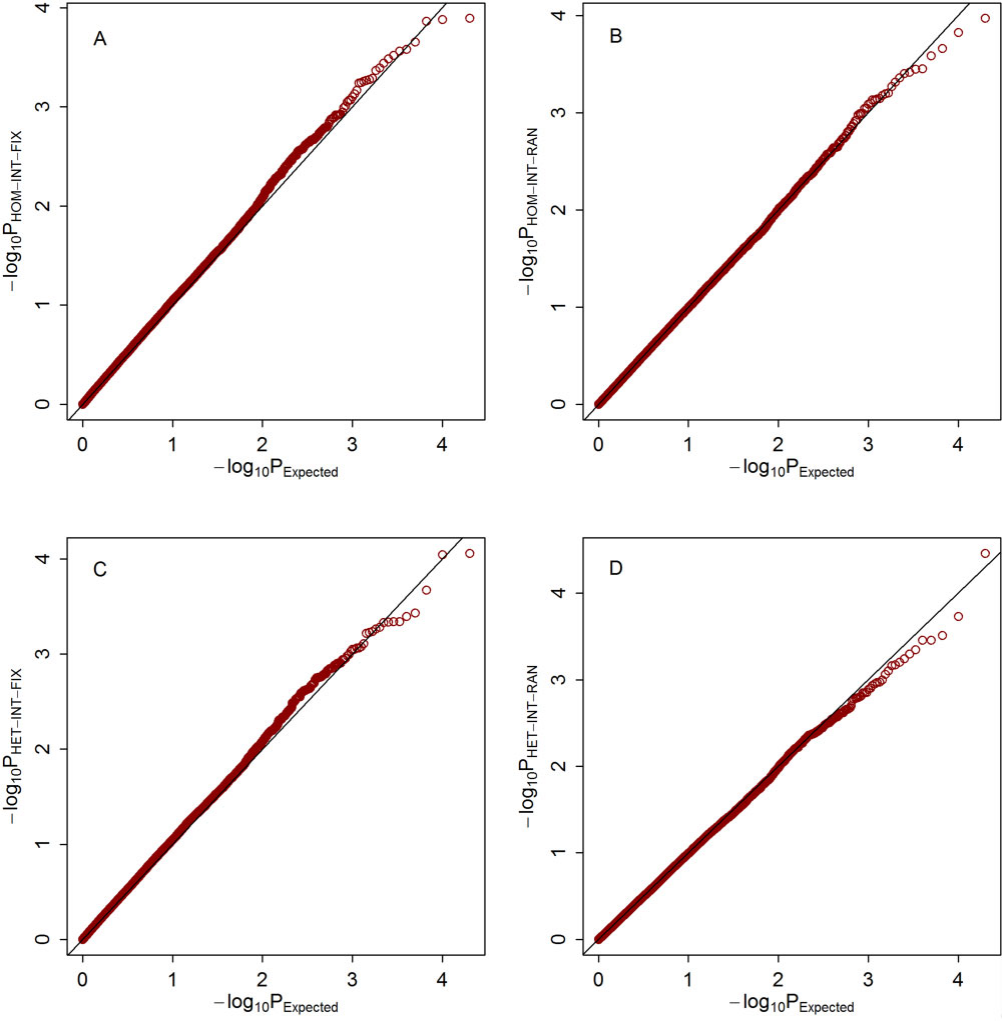
Q–Q plots of the null distributions of the meta-statistics under scenario 3. The horizontal axis represents the negative log_10_ of the expected *P*-values, and the vertical axis represents the negative log_10_ of the observed *P*-values. (A) HOM-INT-FIX; (B) HOM-INT-RAN; (C) HET-INT-FIX; (D) HET-INT-RAN.

Quantile–quantile (Q–Q) plots of the meta-analysis statistics for testing G × E effects under the three scenarios are shown in Figures 1–3, respectively. It can be observed that under all of the three scenarios, the empirical distributions of our four proposed statistics match well with their expected theoretical distributions.

### Statistical power of the meta-statistics

**Figure 4:**
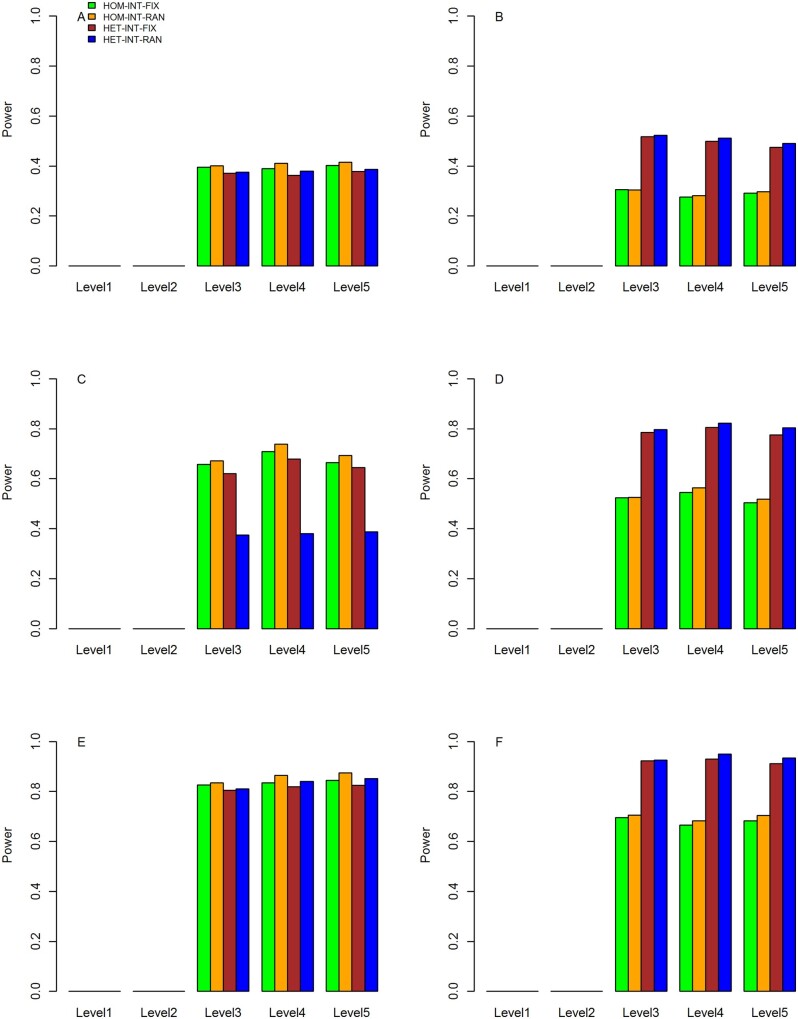
Statistical power of meta-analyses with five levels of genetic main and interaction effects and three proportions of causal rare variants under scenario 1. The horizontal axis represents the level of genetic main and interaction effects, and the vertical axis represents the statistical power. (A) Power of HOM-INT-FIX, HOM-INT-RAN, HET-INT-FIX, and HET-INT-RAN with homogeneous interaction effects across studies and 20% causal rare variants. (B) Power of HOM-INT-FIX, HOM-INT-RAN, HET-INT-FIX, and HET-INT-RAN with heterogeneous interaction effects across studies and 20% causal rare variants. (C) Power of HOM-INT-FIX, HOM-INT-RAN, HET-INT-FIX, and HET-INT-RAN with homogeneous interaction effects across studies and 40% causal rare variants. (D) Power of HOM-INT-FIX, HOM-INT-RAN, HET-INT-FIX, and HET-INT-RAN with heterogeneous interaction effects across studies and 40% causal rare variants. (E) Power of HOM-INT-FIX, HOM-INT-RAN, HET-INT-FIX, and HET-INT-RAN with homogeneous interaction effects across studies and 60% causal rare variants. (F) Power of HOM-INT-FIX, HOM-INT-RAN, HET-INT-FIX, and HET-INT-RAN with heterogeneous interaction effects across studies and 60% causal rare variants.

**Figure 5:**
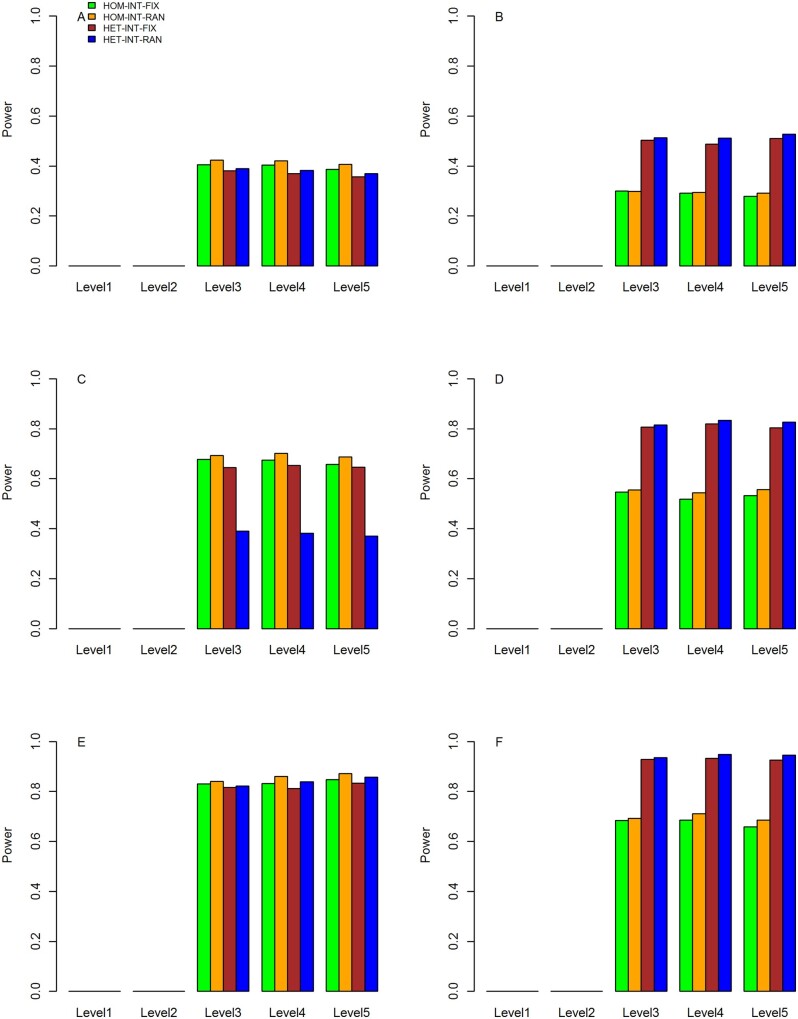
Statistical power of meta-analyses with five different levels of genetic main and interaction effects and three proportions of causal rare variants under scenario 2. The horizontal axis represents the level of genetic main and interaction effects, and the vertical axis represents the statistical power. (A) Power of HOM-INT-FIX, HOM-INT-RAN, HET-INT-FIX, and HET-INT-RAN with homogeneous interaction effects across studies and 20% causal rare variants. (B) Power of HOM-INT-FIX, HOM-INT-RAN, HET-INT-FIX, and HET-INT-RAN with heterogeneous interaction effects across studies and 20% causal rare variants. (C) Power of HOM-INT-FIX, HOM-INT-RAN, HET-INT-FIX, and HET-INT-RAN with homogeneous interaction effects across studies and 40% causal rare variants. (D) Power of HOM-INT-FIX, HOM-INT-RAN, HET-INT-FIX, and HET-INT-RAN with heterogeneous interaction effects across studies and 40% causal rare variants. (E) Power of HOM-INT-FIX, HOM-INT-RAN, HET-INT-FIX, and HET-INT-RAN with homogeneous interaction effects across studies and 60% causal rare variants. (F) Power of HOM-INT-FIX, HOM-INT-RAN, HET-INT-FIX, and HET-INT-RAN with heterogeneous interaction effects across studies and 60% causal rare variants.

**Figure 6:**
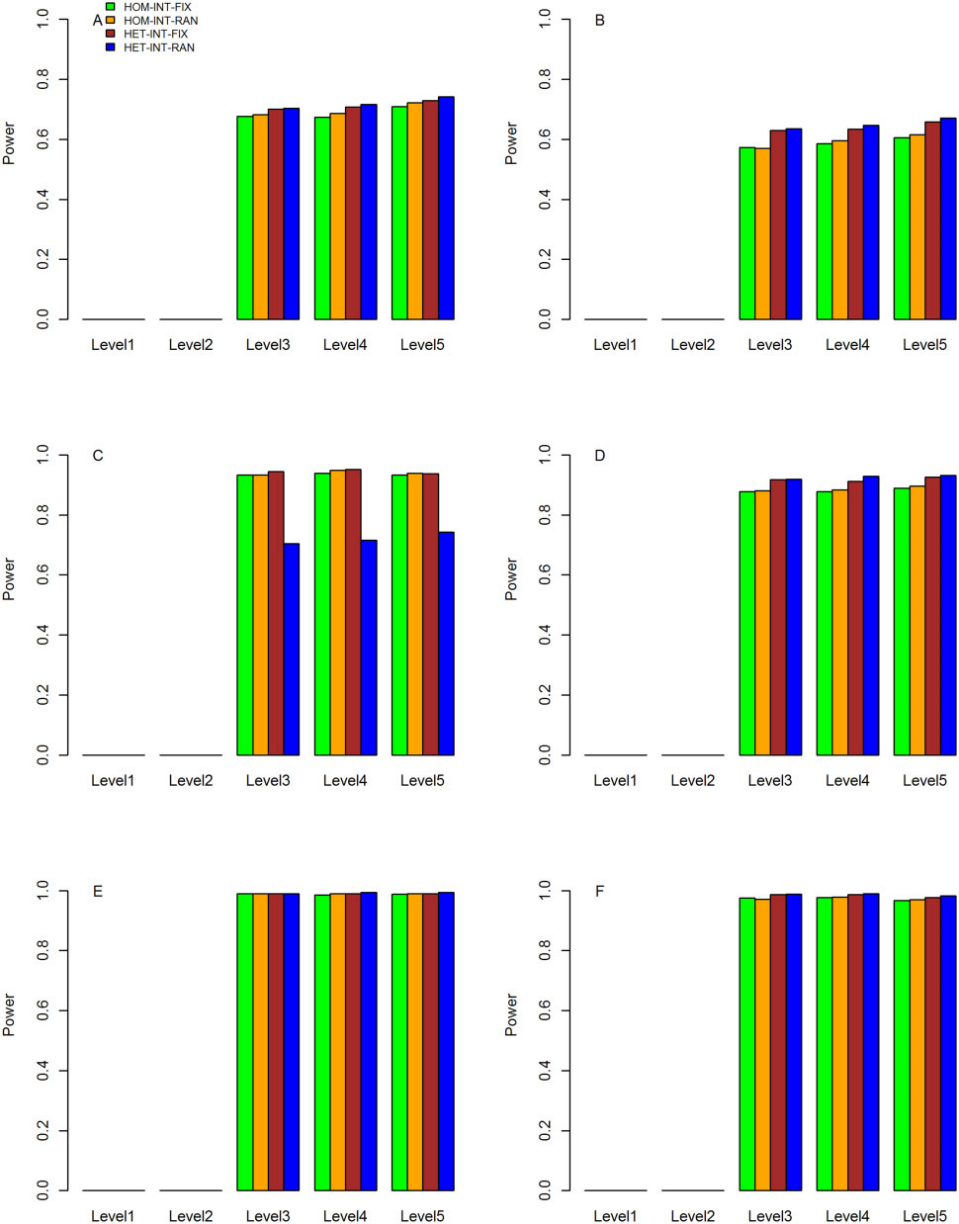
Statistical power of meta-analyses with five levels of genetic main and interaction effects and three proportions of causal rare variants under scenario 3. The horizontal axis represents the level of genetic main and interaction effects, and the vertical axis represents the statistical power. (A) Power of HOM-INT-FIX, HOM-INT-RAN, HET-INT-FIX, and HET-INT-RAN with homogeneous interaction effects across studies and 20% causal rare variants. (B) Power of HOM-INT-FIX, HOM-INT-RAN, HET-INT-FIX, and HET-INT-RAN with heterogeneous interaction effects across studies and 20% causal rare variants. (C) Power of HOM-INT-FIX, HOM-INT-RAN, HET-INT-FIX, and HET-INT-RAN with homogeneous interaction effects across studies and 40% causal rare variants. (D) Power of HOM-INT-FIX, HOM-INT-RAN, HET-INT-FIX, and HET-INT-RAN with heterogeneous interaction effects across studies and 40% causal rare variants. (E) Power of HOM-INT-FIX, HOM-INT-RAN, HET-INT-FIX, and HET-INT-RAN with homogeneous interaction effects across studies and 60% causal rare variants. (F) Power of HOM-INT-FIX, HOM-INT-RAN, HET-INT-FIX, and HET-INT-RAN with heterogeneous interaction effects across studies and 60% causal rare variants.

The results for the statistical power under three scenarios are shown in Figures 4–6, respectively, which include results from five levels of main and interaction effects and three proportions of causal variants. In each figure, the power values on the left are computed based on the simulated data sets in which the variant and gene–BMI interaction effects were the same across studies. The results on the right are based on the data sets with heterogeneous variant and gene–BMI interaction effects across studies.

As can be seen that the statistical powers of the four proposed meta-statistics are close to 0 for interaction effects of levels 1 and 2 where there are no or weak interaction effects. For the interaction effects of levels 3–5, the powers are approximately the same while keeping meta statistics, simulation scenarios and proportions of causal variants fixed. Power results under scenario 1 are almost the same as scenario 2, which suggests that meta-analyses of studies with different covariates distributions had little influence on the power. This is because that the INT-FIX and INT-RAN statistics at study level are essentially based on the phenotypic residuals after adjusting the covariates. Statistical powers under scenario 3 are in general greater than those under scenario 1 or 2, which is due to that AA samples are included and average number of causal variants is larger than that under scenario 1 or 2.

It can be observed that HOM-INT-FIX and HOM-INT-RAN have similar power, and the power of HOM-INT-RAN is slightly greater than that of HOM-INT-FIX. Similar observations can be made for HET-INT-FIX and HET-INT-RAN. This is because INT-FIX and INT-RAN have almost the same power at the study level, which was demonstrated by Chen *et al.* (2014). When the simulated data sets are of homogeneous interaction effects, we can see from the left panels of Figures 4 and 5 that HOM-INT-FIX and HOM-INT-RAN have larger power than those of HET-INT-FIX and HET-INT-RAN. For instance, under scenario 1 with interaction effects of level 3, the power of HOM-INT-RAN is 0.401, 0.671, and 0.834 for 20, 40, and 60% of causal variants, respectively, which are 0.375, 0.637, and 0.811 for HET-INT-RAN. Therefore, HOM-INT-FIX and HOM-INT-RAN are preferable to HET-INT-FIX and HET-INT-RAN for meta-analyses of studies that are highly comparable, for which the underlying interaction effects are likely to be homogeneous.

Under scenario 3, we can see from the left panels of Figure 6 that HET-INT-FIX and HET-INT-RAN have slightly greater or nearly the same power as HOM-INT-FIX and HOM-INT-RAN because of the heterogeneous effects between EUR and AA samples. When the simulated data sets are based on heterogeneous variant and gene–BMI interaction effects across studies, as can be depicted from the right panels of Figures 4–6 that HET-INT-FIX and HET-INT-RAN have larger power than those of HOM-INT-FIX and HOM-INT-RAN for the same proportion of causal variants under all scenarios. For instance, under scenario 1 with interaction effects of level 3, HOM-INT-RAN has power values of 0.304, 0.526, and 0.705 for three proportions of causal variants, respectively, while HET-INT-RAN has corresponding power values of 0.523, 0.797, and 0.925. Not surprisingly, HET-INT-FIX and HET-INT-RAN are superior to HOM-INT-FIX and HOM-INT-RAN in this case, since the interaction effects are heterogeneous across studies in the simulated data sets and the former two approaches appropriately account for such heterogeneity.

**Figure 7:**
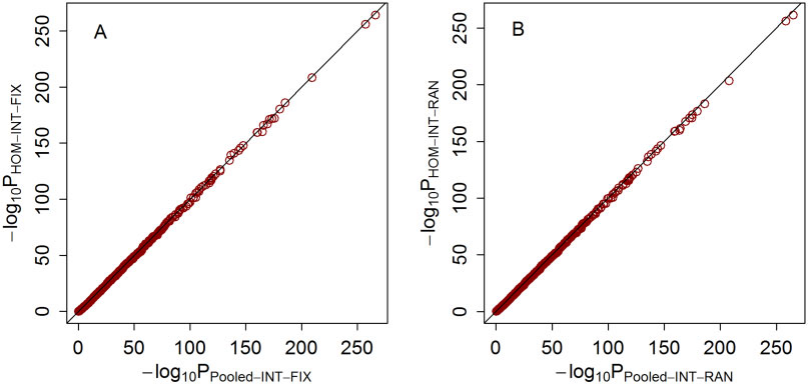
*P*-values of HOM-INT-FIX, HOM-INT-RAN, Pooled-INT-FIX and Pooled-INT-RAN with homogeneous interaction effects across studies, interaction effects of level 3 and 40% causal rare variants under scenario 1. (A) *P*-values of HOM-INT-FIX and Pooled-INT-FIX. The horizontal axis represents the negative log_10_*P*-values of Pooled-INT-FIX, and the vertical axis represents the negative log_10_*P*-values of HOM-INT-FIX. (B) *P*-values of HOM-INT-RAN and Pooled-INT-RAN. The horizontal axis represents the negative log_10_*P*-values of Pooled-INT-RAN, and the vertical axis represents the negative log_10_*P*-values of HOM-INT-RAN.

To evaluate the statistical efficiency of the proposed meta-analysis methods, we compared the statistical power of HOM-INT-FIX and HOM-INT-RAN with that of pooled analyses conducted based on interaction tests with individual-level data from all studies under scenario 1. The results with 40% causal variants, interaction effects of level 3 and homogeneous interaction effects across studies are shown in Figure 7. The *P*-values of the HOM-INT-FIX meta-analysis and the pooled analysis with the INT-FIX test are compared in Figure 7A. As we can see, almost all points lie on a diagonal line, indicating that the HOM-INT-FIX meta-analysis is equally as powerful as the single pooled interaction test based on INT-FIX. Similarly, Figure 7B shows that the HOM-INT-RAN meta-analysis is equally as powerful as the pooled INT-RAN analysis. Because the pooled INT-FIX and INT-RAN analyses implicitly assume the same interaction effect across studies, these results demonstrate that there is no power loss with the HOM-INT-FIX and HOM-INT-RAN meta-analyses when this assumption is satisfied.

**Figure 8:**
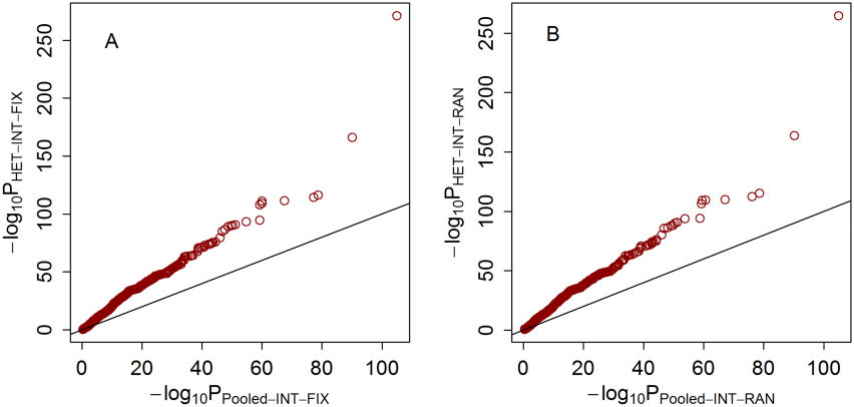
*P*-values of HET-INT-FIX, HET-INT-RAN, Pooled-INT-FIX, and Pooled-INT-RAN with heterogeneous interaction effects across studies, interaction effects of level 3 and 40% causal rare variants under scenario 1. (A) *P*-values of HET-INT-FIX and Pooled-INT-FIX. The horizontal axis represents the negative log_10_*P*-values of Pooled-INT-FIX, and the vertical axis represents the negative log_10_*P*-values of HET-INT-FIX. (B) *P*-values of HET-INT-RAN and Pooled-INT-RAN. The horizontal axis represents the negative log_10_*P*-values of Pooled-INT-RAN, and the vertical axis represents the negative log_10_*P*-values of HET-INT-RAN.

We also compared the statistical power of the HET-INT-FIX and HET-INT-RAN meta-analyses with that of the pooled INT-FIX and INT-RAN analyses, respectively. For scenario 1 with 40% causal variants, interaction effects of level 3 and heterogeneous interaction effects across studies, the results are shown in Figure 8. As we can see, the *P*-values of the HET-INT-FIX and HET-INT-RAN meta-analyses are smaller than those of the pooled INT-FIX and INT-RAN analyses. Therefore, HET-INT-FIX and HET-INT-RAN are more powerful than the pooled analyses when heterogeneity of the interaction effect exists. This is because the HET-INT-FIX and HET-INT-RAN meta-analyses treat genetic heterogeneity appropriately when synthesizing the summary results from multiple studies. By contrast, the pooled individual-level data contain mixed interaction effects for the same variants, thus violating the underlying assumptions of INT-FIX and INT-RAN.

### Gene–age interactions in blood pressure traits with UK Biobank data

UK Biobank is a prospective cohort study involving approximately 500,000 volunteers in the United Kingdom aged between 40 and 69 years, from whom extensive genetic and phenotypic data have been collected (Sudlow *et al.* 2015; Bycroft *et al.* 2018). The latest release of whole-exome sequencing data contains data from 200,643 participants. We extracted participants with European, African or Asian ancestry and excluded participants who had withdrawn and one member of each pair of first- or second-degree relatives. The average SBP and diastolic blood pressure (DBP) measurements at the first visit were used. For participants taking antihypertensive medications at the time of the visit, 10 and 5 mm Hg were added to their SBP and DBP measurements, respectively (Cui *et al.* 2003). Mean arterial pressure (MAP) and pulse pressure (PP) were calculated as MAP=SBP/3+2DBP/3 and PP=SBP-DBP, respectively, and the latter was then logarithmically transformed. Participants with missing BP phenotypes or covariates, including age, BMI and sex, were excluded. Phenotype and covariate outliers were also excluded, where these were defined as data points lying at least 5 standard deviations away from the corresponding means. In summary, a total of 162,148 European samples, 3113 African samples and 4745 Asian samples with complete phenotype, covariates, and exome genotype data were included in our analyses.

To conduct meta-analyses of multiple studies of the same ancestry, we divided the European samples into five groups based on the geographic locations of their assessment centers: group 1 included participants from assessment centers in Edinburgh, Glasgow, Middlesbrough, and Newcastle; group 2 was from Barts, Croydon, Hounslow, Oxford, and Reading; group 3 was from Bristol, Cardiff, and Swansea; group 4 was from Leeds, Sheffield, Nottingham, and Birmingham; and group 5 was from Bury, Cheadle, Liverpool, Manchester, Stockport, Stoke, and Wrexham. For trans-ethnic meta-analyses, group 6 of African samples and group 7 of Asian samples were included. The characteristics of samples in the seven groups are presented in Table 2.

**Table 2:** Characteristics of UK Biobank samples in seven groups

Group	*N*	Male (%)	HTx (%)	Age (years)		BMI (kg/m^2^)		SBP (mmHg)		DBP (mmHg)	
				Mean	SD	Mean	SD	Mean	SD	Mean	SD
1	27,154	44.0	21.4	56.8	8.0	27.5	4.7	139.7	18.8	83.2	10.1
2	31,282	44.5	18.4	57.0	8.0	26.7	4.6	135.3	18.0	81.1	10.0
3	18,932	43.5	18.9	56.1	8.2	27.3	4.7	139.3	18.5	83.1	10.0
4	52,721	46.0	20.7	57.1	7.9	27.5	4.7	138.3	18.4	82.2	10.0
5	32,059	47.0	21.5	56.9	7.9	27.6	4.7	138.4	18.4	82.0	10.0
6	3,113	41.5	31.9	52.0	8.0	29.5	5.3	138.0	18.6	84.9	10.7
7	4,745	50.0	22.9	53.0	8.4	26.7	4.3	134.3	18.6	82.5	10.3

N, number of participants; HTx, percentage of individuals on antihypertensive medications at the time of clinic visit; BMI, body mass index; DBP, diastolic blood pressure; SBP, systolic blood pressure; SD, standard deviation.

We selected nine loci that showed nominal evidence (*P* < 0.05) of SNP–age interaction in a genome-wide search of common variants with age-dependent effects in BP regulation (Simino *et al.* 2014). For the reported leading SNPs that are in gene regions, the corresponding genes were selected for analyzing gene–age interaction with rare variants; otherwise, the nearest up- and downstream genes were chosen. In total, 12 candidate genes were selected from the nine loci. The variants of the 12 genes were annotated with VEP (McLaren *et al.* 2016), and those annotated as stop_loss, missense_variant, start_lose, splice_donor_variant, inframe_deletion, frameshift_variant, splice_acceptor_variant, stop_gained, or inframe_insertion were used for analysis. In addition, variants that were PolyPhen or SIFT benign, defined as variants with PolyPhen scores smaller than 0.15 or SIFT scores larger than 0.05 (Cirulli *et al.* 2020), were excluded. PLINK (Chang *et al.* 2015) was used to extract rare variants with MAFs smaller than 0.03, and fcGENE (Roshyara and Scholz 2014) was used to convert genotypes into numeric values. The numbers of variants in the genes used for the single and multiple ancestries meta-analyses are shown in Tables 3 and 4, respectively.

**Table 3:** P-values of meta-analyses and pooled analyses of gene–age interaction in BP traits for the five geographic groups of European ancestry in the UK Biobank data

Loci	SNP	Up/down stream gene	RV Num	Trait	*P*-value					
					HOM-INT-FIX	HOM-INT-RAN	HET-INT-FIX	HET-INT-RAN	Pooled-INT-FIX	Pooled-INT-RAN
1	rs880315	*CASZ1*	145	SBP	0.95	0.96	0.63	0.67	0.94	0.93
2	rs6797587	*CDC25A*	25	MAP	0.03	0.06	0.08	0.10	0.04	0.05
3	rs11099098	*PRDM8*	59	SBP	0.82	0.92	0.93	0.96	0.88	0.92
		*FGF5*	7	SBP	0.41	0.52	0.24	0.30	0.50	0.46
4	rs198846	*HIST1H1T*	71	DBP	0.18	0.19	0.27	0.25	0.17	0.19
5	rs12705390	*CCDC71L*	7	PP	0.70	0.95	0.85	0.96	0.60	0.94
		*PIK3CG*	65	PP	0.82	0.79	0.40	0.48	0.76	0.73
6	rs7070797	*C10orf107*	19	MAP	0.07	0.06	0.04	0.13	0.06	0.10
		*ARID5B*	60	MAP	0.94	0.95	0.74	0.94	0.89	0.91
7	rs4601790	*EHBP1L1*	74	MAP	0.05	0.06	0.11	0.10	0.07	0.06
8	rs11072518	*COX5A*	16	MAP	0.54	0.55	0.65	0.66	0.56	0.57
9	rs17608766	*GOSR2*	12	PP	0.22	0.20	0.69	0.71	0.20	0.15

RV Num, number of rare variants; SBP, systolic blood pressure; DBP, diastolic blood pressure; MAP, mean arterial pressure; PP, pulse pressure.

**Table 4:** P-values of meta-analyses and pooled analyses of gene–age interaction in BP traits for the seven groups of African, Asian, and European ancestries in the UK Biobank data

Loci	SNP	Up/down stream gene	RV Num	Trait	*P*-value					
					HOM-INT-FIX	HOM-INT-RAN	HET-INT-FIX	HET-INT-RAN	Pooled-INT-FIX	Pooled-INT-RAN
1	rs880315	*CASZ1*	164	SBP	0.96	0.97	0.95	0.96	0.93	0.93
2	rs6797587	*CDC25A*	28	MAP	0.03	0.06	0.03	0.06	0.05	0.05
3	rs11099098	*PRDM8*	66	SBP	0.83	0.88	0.83	0.94	0.84	0.88
		*FGF5*	7	SBP	0.41	0.52	0.41	0.52	0.52	0.47
4	rs198846	*HIST1H1T*	85	DBP	0.24	0.26	0.19	0.21	0.19	0.13
5	rs12705390	*CCDC71L*	7	PP	0.95	0.99	0.95	0.98	0.87	0.99
		*PIK3CG*	73	PP	0.82	0.79	0.86	0.83	0.82	0.75
6	rs7070797	*C10orf107*	24	MAP	0.15	0.13	0.10	0.09	0.24	0.35
		*ARID5B*	64	MAP	0.87	0.94	0.88	0.94	0.75	0.95
7	rs4601790	*EHBP1L1*	91	MAP	0.05	0.05	0.06	0.06	0.02	0.03
8	rs11072518	*COX5A*	16	MAP	0.54	0.58	0.54	0.56	0.64	0.54
9	rs17608766	*GOSR2*	13	PP	0.43	0.44	0.39	0.37	0.45	0.45

RV Num, number of rare variants; SBP, systolic blood pressure; DBP, diastolic blood pressure; MAP, mean arterial pressure; PP, pulse pressure.

For each of the 12 genes, we first conducted INT-FIX and INT-RAN analyses on each of the seven groups with the primary BP traits reported in Simino *et al.* (2014) and obtained summary results. Age, sex, BMI, and the first 10 principal components were used as covariates, and age was used as the “environmental” variable. The summary results from the five groups of European ancestry were then combined by means of HOM-INT-FIX, HOM-INT-RAN, HET-INT-FIX, and HET-INT-RAN. For comparison, we performed INT-FIX and INT-RAN analyses on all samples of the five groups, denoted by Pooled-INT-FIX and Pooled-INT-RAN. The *P*-values of the four meta-analyses and the two pooled analyses are displayed in Table 3. For the trans-ethnic meta-analyses based on summary results from the seven groups, we also conducted the four meta-analyses: HOM-INT-FIX, HOM-INT-RAN, HET-INT-FIX, and HET-INT-RAN, and the Pooled-INT-FIX and Pooled-INT-RAN, whose *P*-values are displayed in Table 4.

Similar to the simulation results, HOM-INT-FIX and HOM-INT-RAN show approximately the same *P*-values, and the *P*-values of HET-INT-FIX and HET-INT-RAN are close in Tables 3 and 4. HOM-INT-FIX yields *P*-values approximately equal to those of Pooled-INT-FIX, and HOM-INT-RAN has roughly the same *P*-values as Pooled-INT-RAN in Table 3. Moreover, HOM-INT-FIX and HOM-INT-RAN have smaller *P*-values than HET-INT-FIX and HET-INT-RAN for most of the 12 genes. This suggests that the interaction effects, if they are genuine, are homogeneous across the five groups of European ancestry in UK Biobank. Two genes show nominal evidence of interaction with age in the meta-analyses. *CDC25A* and *C10orf107* have *P*-values of 0.03 and 0.04 in HOM-INT-FIX and HET-INT-FIX, respectively. After correction for multiple testing, these results are no longer significant.

In Table 4, the *P*-values of HOM-INT-FIX are larger than those of HET-INT-FIX, and the *P*-values of HOM-INT-RAN are larger than those of HET-INT-RAN, which suggests that the interaction effects, if they exist, are heterogeneous across ancestry groups in the UK Biobank data. Besides, the *P*-values of Pooled-INT-FIX are larger than those of HET-INT-FIX, which also indicates that the interaction effects are heterogeneous. *CDC25A* has *P*-value of 0.03 in both HOM-INT-FIX and HET-INT-FIX, which are no longer significant after the correction for multiple testing.

## Discussion

In this study, we propose four meta-analysis methods for testing G × E effects with rare variants while treating main genetic effects as either fixed or random and considering homogeneous or heterogeneous G × E effects across studies. Simulations as well as an analysis of UK Biobank data demonstrate that treating variant main effects as either fixed or random provides approximately the same statistical power. The results are consistent with the conclusion in Chen *et al.* (2014) that INT-FIX has almost the same power as INT-RAN when analyzing interaction in a single study. It has been suggested that when the number of genetic variants is large, INT-FIX will lead to unstable estimates of the main genetic effects and, thus, INT-RAN is recommended (Chen *et al.* 2014). Therefore, our HOM-INT-FIX and HET-INT-FIX meta-analyses are valid only if INT-FIX is applicable for the study-level analysis. Otherwise, INT-RAN should be used in the studies, and HOM-INT-RAN or HET-INT-RAN should be used for the meta-analysis.

We developed meta-analyses for testing G × E effects by combining summary results from INT-FIX and INT-RAN (Chen *et al.* 2014) at study level based on the analysis framework proposed by Lee *et al.* (2013). The methods offer much larger sample size for testing the interaction which is impossible for a single study. They were shown to be statistical efficient in our simulation studies as well as the analyses of UK Biobank data. It is well established that for the meta-analysis of association statistics with common variants, the power loss is minimal (Lin and Zeng 2010). However, the power loss of meta-analysis for rare variants is largely unexamined and has been suspected to be possibly more sizable (Panagiotou *et al.* 2013). In this study, we have compared the HOM-INT-FIX and HOM-INT-RAN meta-analyses with pooled analyses based on INT-FIX and INT-RAN, respectively. Our results show that they have approximately the same power. In gene–age interaction analyses of UK Biobank data, the results from the meta-analyses and the pooled analyses are close. Therefore, the power loss of meta-analysis for rare variants is concluded to be minimal as well.

In the gene–age interaction analysis of UK Biobank data, none of the 12 genes from the nine candidate loci shows experiment-wide significant results. There are many possible reasons. First, most GWAS SNPs are from noncoding regions, and some of them may play regulatory roles by changing the expression levels of the modulated genes (Edwards *et al.* 2013). Nevertheless, the rare variants selected in our analyses likely alter the protein sequences that the genes express, which are not necessarily correlated with the regulatory SNPs. Second, it has been estimated that only approximately one-third of causal genes are the nearest genes to the GWAS loci (Gusev *et al.* 2016; Zhu *et al.* 2016). Thus, we may have missed the majority of the causal genes, which are located farther away from the candidate loci. Finally, the nine loci identified in the genome-wide analysis show only nominal evidence of interactions, which are not genome-wide significant. Therefore, some of the loci considered in the analyses may represent spurious interaction effects.

In this study, we have demonstrated the proposed meta-analyses for continuous traits. The methods can be readily generalized to the case of binary traits. For binary traits, logistic versions of INT-FIX and INT-RAN can be applied at the study level, and the G × E score statistics for single variants and the relationship matrices can be computed based on (5) and (7)–(9), the same formulas as for continuous traits. There is no difference in how the meta-statistics are computed for binary and continuous traits. However, when studies have unbalanced case-control ratios and minor allele counts in a gene are very low, using saddlepoint approximation can result in inaccurate *P*-values for binary traits, and efficient resampling can be used instead (Lee *et al.* 2016).

Our proposed meta-analyses are limited to testing G × E effects only. Joint testing of main genetic effects and interaction effects has long been suggested for the interaction analysis of common variants (Kraft *et al.* 2007; Manning *et al.* 2011). Joint testing offers better power than the analysis of main genetic effects only and the analysis of interaction effects only when both types of effects exist. In future work, we will further extend our meta-analyses to allow the joint testing of main genetic effects and G × E effects.

## Conclusions

In this study, we proposed four powerful meta-analysis methods for testing G × E effects with rare variants. We considered both homogeneous G × E effects and heterogeneous G × E effects across studies and efficiently combined the summary statistics of INT-FIX and INT-RAN from multiple studies. Through simulations and real data analysis, we demonstrated that our approaches provide power comparable to that of pooled analysis when the interaction effects are homogeneous across studies. When heterogeneity exists across studies, our approaches can treat heterogeneity appropriately and achieve greater statistical power than a pooled analysis. Our meta-analysis methods of testing G × E effects can be applied to synthesize results from multiple diverse studies to increase the effective sample size and improve the chance of identifying genes whose effects are modified by an environmental factor.

## Funding

This work was supported by the national Thousand Youth Talents Plan.
